# Knowledge, Attitudes, Practices, and Information Pathways Related to Brucellosis Among Adults in Najran City, Saudi Arabia: A Stratified Time–Location Cross-Sectional Study

**DOI:** 10.3390/tropicalmed11060149

**Published:** 2026-05-29

**Authors:** Abdullateef Abdullah Alshehri, Mohammad Y. Alqahtani, Osman AE. Elnoubi, Mohsen A. Qahtani, Dehiyyan E. Alyami, Meshal M. Alabbas, Mosa M. Bahnass, Abdullah Alshehari, Mohammed A. Alshehri, Mohammed A. Alshahrani

**Affiliations:** 1 Department of Clinical Laboratory Sciences, Faculty of Applied Medical Sciences, Najran University, P.O. Box 1988, Najran 61441, Saudi Arabiamaalshahrani@nu.edu.sa (M.A.A.); 2 Department of Infection Prevention and Control, Najran University Hospital, Najran University, P.O. Box 1988, Najran 61441, Saudi Arabia; dealyami@nu.edu.sa; 3 Department of Community, Psychiatric and Mental Health Nursing, Nursing College, Najran University, P.O. Box 1988, Najran 61441, Saudi Arabia; 4 Health Research Center, Najran University, P.O. Box 1988, Najran 61441, Saudi Arabia; 5 Department of Laboratory Medicine, Specialist Laboratory Section, Najran Armed Forces Hospital, P.O. Box 8644, Najran 66252, Saudi Arabia; 6 Project Management Office, Aseer Health Cluster, Quality Office, Khamis Mushait Maternity and Children’s Hospital, Ministry of Health, Aseer Region, P.O. Box 6542, Khamis Mushait 62493, Saudi Arabia; 7 Department of Diagnostic Radiology, Armed Forces Hospital Southern Region, P.O. Box 101, Khamis Mushait 62413, Saudi Arabia; mas3399@hotmail.com

**Keywords:** brucellosis, knowledge, attitudes, and practices (KAP), health communication, One Health, zoonoses, public health, Najran

## Abstract

Brucellosis remains an important zoonotic disease in southern Saudi Arabia; however, community-level knowledge, risk-related practices, and information pathways in Najran City are insufficiently characterized. This study assessed brucellosis-related knowledge, attitudes, practices, and information pathways among adults in Najran City to inform locally relevant One Health interventions. In this cross-sectional survey, adults were recruited using stratified time–location (venue-based) sampling across community and exposure-relevant sites in Najran City. A total of 608 adults completed a structured interviewer-administered questionnaire. Composite scores were calculated for knowledge (0–21), attitude (0–22), practice (0–64), and information-source breadth (0–6). Descriptive statistics, group comparisons, correlation analyses, and multivariable linear regressions were performed. The findings suggest that participants more commonly relied on interpersonal social networks, especially family and friends, for information related to brucellosis (53.9%), whereas formal sources were less commonly reported, including health professionals (7.9%), media (4.6%), internet sources (3.3%), educational institutions (2.0%), and agricultural or veterinary organizations (1.3%). Mean knowledge scores were moderate (10.7/21), attitudes were generally favorable (19.5/22), and practice scores were moderate (36.6/64). Exposure-related behaviors remained common, particularly the consumption of unpasteurized milk or dairy products (56.6%). The breadth of information sources showed a moderate positive correlation with knowledge (rho = 0.561), whereas attitude showed only small positive correlations with knowledge and practice. Finally, knowledge was weakly and inversely correlated with practice. Among adults recruited in this venue-based sample, favorable attitudes did not consistently correspond to safer practices. These findings support practical One Health interventions, including coordinated veterinary–public health messaging on animal abortion events, safe-dairy guidance at points of sale and community venues, workplace-based training for livestock-contact groups, and referral pathways linking suspected animal cases with veterinary services and human care-seeking. Because recruitment was venue-based and non-probability, the results should be interpreted as descriptive and hypothesis-generating rather than population-representative; however, they still identify practical communication and service-delivery priorities for future intervention studies in Najran.

## 1. Introduction

Brucellosis is a globally distributed bacterial zoonosis that remains particularly important in settings characterized by intensive livestock husbandry and frequent human–animal contact [1,2,3,4]. Human infection occurs through direct contact with infected animals, exposure to aborted materials, or ingestion of contaminated animal-derived products, particularly unpasteurized dairy items [5,6]. Human brucellosis often presents as a nonspecific febrile illness with symptoms such as malaise, recurrent fever, arthralgia, myalgia, and back pain. If not promptly recognized and managed, infection may progress to chronic or focal complications that affect quality of life and productivity [6,7]. The greatest burden and highest risk settings are concentrated in Africa and Asia, although the Americas and Europe also face significant concerns regarding the disease [1,2,3]. Human brucellosis is most commonly associated with exposure to infected livestock or contaminated animal products, particularly from sheep, goats, cattle, and camels in endemic settings [4,5,8,9].

Understanding community knowledge, attitudes, and practices (KAPs) is essential to designing effective brucellosis prevention and control interventions. Previous research has shown that inadequate knowledge and unsafe practices are important barriers to disease control [10,11,12]. For example, farmers need to recognize the symptoms of brucellosis in animals, avoid consuming raw milk, and adopt appropriate biosecurity practices [13,14]. In one study, although 70% of farmers were aware of animal brucellosis, only 26% were familiar with the human form of the disease [14]. The same study found that 66% of farmers consumed raw milk, and many did not follow appropriate hygiene procedures during milking, thereby increasing the risk of animal-to-human transmission [14]. Similarly, studies from various settings have reported insufficient understanding of brucellosis transmission pathways and preventive measures [10,13]. These findings indicate that educational interventions must address not only general awareness, but also the specific behaviors that sustain transmission.

Human brucellosis has been investigated across several regions of the Kingdom of Saudi Arabia [15,16,17,18,19,20,21]. It remains among the most frequently reported zoonotic diseases in the country, especially in Riyadh [12,22]. Owning sheep and frequent contact with animals have been identified as major risk factors for brucellosis, which remains highly prevalent among agropastoral communities in southern Saudi Arabia, particularly in the northwest Aseer region [23]. These observations underscore the need for integrated One Health strategies and targeted public health interventions to improve prevention and control [23]. According to national surveillance reports, the incidence of brucellosis in the Najran region was three cases per 10,000 population by the end of 2023 [24].

Cultural norms and household economics can substantially influence risk behaviors associated with brucellosis among livestock-keeping communities. Despite basic awareness of the disease, comparative data from Kenya and Kyrgyzstan demonstrate that deeply rooted beliefs, reliance on traditional healers, and cost-conscious decision-making frequently impede veterinary consultation, diminish vaccination and testing rates, and normalize hazardous practices such as raw-milk consumption, improper disposal of fetal membranes and placentas, and inadequate on-farm biosecurity measures [25,26]. These observations are relevant to Saudi Arabia, including Najran, because mixed urban–peri-urban livestock keeping, informal dairy consumption, household animal-care practices, and livestock movement may create similar interfaces between human behavior, animal health, and food safety. Recent KAP evidence from Kazakhstan provides comparable data form a Central Asian setting, showing higher knowledge among veterinary professionals than farmers but persistent knowledge–practice gaps related to human health risks, aborted material handling, raw milk consumption, and disposal of reproductive tissues [27]. Furthermore, awareness-raising initiatives underperform if constraints such as affordability, the gendered division of labor and decision-making authority, and the role of social sanctions in supporting behavioral change are unaccounted for [12,13]. Therefore, a context-specific study is needed to identify locally relevant beliefs, map information sources and decision-making processes, and understand occupational risk behaviors. These findings could inform co-developed One Health initiatives—such as safe-milk messaging, safe management guidance for animal abortion events, community-endorsed waste disposal practices, and practical biosecurity protocols—implemented through collaboration across animal health, environmental health, public health, and community stakeholders to ensure they are culturally appropriate, cost-effective, and scalable.

Many brucellosis surveys have relied on venue-based convenience samples and broad occupational groupings, which is a design choice that can weaken external validity and limit the practical value of their recommendations. Accordingly, context-specific KAP evidence from animal-producing and agriculture-focused settings remains important for identifying high-risk practices, barriers to service uptake, and locally trusted information channels. Such evidence can help inform region-specific strategies for risk communication, biosecurity, and vaccination [28,29,30,31].

Najran is a southern border province, known for transboundary livestock movement, smallholder dairy farming, and production and consumption of raw milk. Previous research on brucellosis in Najran has mainly focused on diagnosis, epidemiology, and candidate clinical or laboratory indicators. One study highlighted the value of combining serological and molecular diagnostic methods, which may improve the detection and clinical management of human brucellosis [32]. Furthermore, studies have identified distinct patterns in pediatric cases, underscoring the importance of targeted public health interventions to mitigate exposure in this vulnerable demographic [33]. Other studies reported that selected biochemical and hematological markers may support earlier recognition of brucellosis, which could be useful in resource-limited settings [34]. While these contributions provide a useful foundation for developing effective control strategies for brucellosis in endemic regions such as Najran, a significant gap persists. Prior work from Najran has documented serological evidence of brucellosis in both human and animal samples in the region [35]. However, detailed information remains limited regarding community knowledge, preventive attitudes, and everyday practices relevant to brucellosis transmission in Najran. This gap provides the rationale for the present study, which seeks to move beyond clinical and laboratory descriptions by systematically characterizing community knowledge, attitudes, practices, and information pathways relevant to brucellosis prevention in Najran. By examining information sources alongside KAP scores, the study aims to generate practice-relevant evidence to support change in behavior, biosafety messaging, and future surveillance-oriented work. Unlike previous studies conducted in Najran, which predominantly focused on clinical diagnosis, epidemiology, or laboratory indicators, this research offers a systematic evaluation of community KAPs, occupationally relevant risk behaviors, and information dissemination channels within a mixed urban–peri-urban smallholder setting.

Thus far, knowledge, risk perceptions, and routine practices among high-exposure groups such as smallholder farmers, abattoir workers, and household livestock keepers have not been systematically assessed. This knowledge gap is particularly important because it shapes how individuals respond to events such as calving or abortion, including the use of protective measures, the disposal of abortive materials, and care-seeking when experiencing febrile illness compatible with brucellosis. These practices can substantially influence both exposure risk and opportunities for earlier disease detection [36,37,38]. Consequently, we conducted a KAP assessment, focusing on occupational patterns in Najran. We employed stratified time–location (venue-based) recruitment and a structured questionnaire adapted from prior KAP instruments [11,13,14,19,20,21,39]. The objectives were to describe locally relevant misconceptions and behaviors, identify trusted information sources, and inform One Health risk communication and biosecurity messaging around animal parturition and abortion events, as well as timely and appropriate care-seeking for suspected infection. Behavioral endpoints were prespecified to capture knowledge deficits and high-risk practices across the main transmission routes, thereby identifying modifiable gaps relevant to intervention design and future representative studies. In this study, the One Health perspective is operationalized by linking KAP and information-source findings to coordinated action across human health services, animal health and food-system sectors, as well as community settings such as educational institutions, markets, and digital platforms.

This study describes knowledge, attitudes, practices, and information pathways related to brucellosis among adults recruited through stratified time–location (venue-based) sampling in an urban–peri-urban area of Najran City. We examine associations between demographic and occupational characteristics, information sources, and KAP scores to support hypothesis generation and refine target populations for future representative surveys and interventions. Because recruitment was based on venue rather than household probability, findings within the recruited sample are interpreted as descriptive, association-based, and hypothesis-generating; key patterns should be confirmed in probability-based surveys before any population-level inferences are made for Najran City.

## 2. Materials and Methods

### 2.1. Study Design and Sample Size

**Figure 1:**
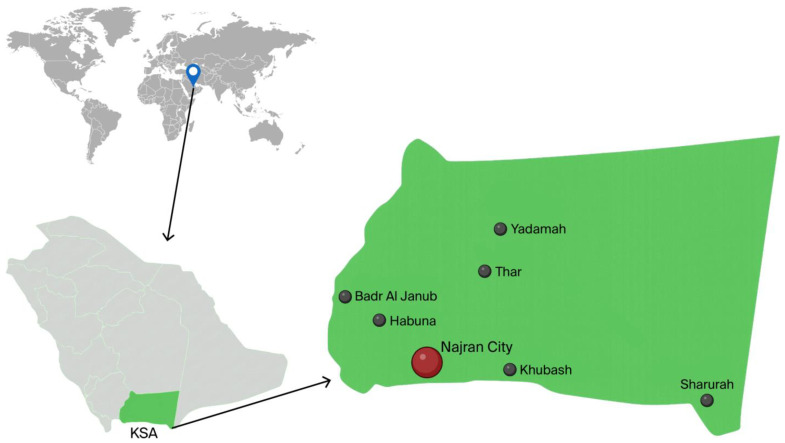
Location of Najran City within Najran Province, Saudi Arabia.

To assess brucellosis-related KAPs, we conducted a cross-sectional survey in Najran City, Kingdom of Saudi Arabia, from June 2024 to May 2025 (Figure 1). The target population comprised adult residents aged 18 years or older in Najran City, including community members and individuals in occupations with potential exposure to livestock or animal products, such as smallholder dairy farmers, slaughterhouse workers, and veterinarians.

Participants were recruited using stratified time–location (venue-based) sampling. Venue-time blocks were defined across neighborhood clusters and public or exposure-relevant locations, including municipal markets, dairy outlets, urban livestock holdings, community centers, Najran University, and Najran Armed Forces Hospital. This approach was selected to improve geographic coverage across the city, reach both general community members and higher-exposure occupational groups, and capture behaviors relevant to the transmission of brucellosis in the absence of a complete household sampling frame. Because individual probabilities of selection were not known, the final sample should be considered a non-probability sample. Accordingly, the findings are interpreted as descriptive of the recruited sample and association-based rather than representative of the entire adult population in Najran City.

The 2022 census population of Najran City (N = 381,431) defined the geographic target population for planning purposes [40]. The minimum sample size was planned a priori using the single-proportion formula with a 95% confidence level, a conservative expected proportion of 50%, and a precision of 4%, resulting in a target sample of approximately 600 participants [41]. Because the target population was finite (N = 381,431), a finite population correction was considered during planning; the corrected estimate remained close to 600 and was therefore retained as the prespecified minimum sample size. The planned sample size was used to support analytic stability and descriptive precision within the recruited sample. A total of 643 individuals were approached, of whom 608 met the inclusion criteria and completed the questionnaire.

### 2.2. Questionnaire Development and Interviewer Training

Data were collected using a structured interviewer-administered questionnaire designed to assess demographic characteristics and knowledge, attitudes, practices, and information pathways related to brucellosis among participants. The questionnaire was adapted from prior brucellosis KAP methods used among livestock owners, smallholder dairy farmers, community populations, and occupationally exposed groups [11,13,14,19,20,21,39]. Items were reviewed by the study team for local relevance to Najran, focusing on raw dairy consumption, contact with livestock, animal abortion management, care-seeking, and information pathways. The adapted questionnaire was reviewed for clarity, face validity, and content relevance before field implementation. Interviewers were trained in a standardized participant approach, with neutral question delivery, response recording, and confidentiality procedures. Occupation was assigned according to the participant’s main current role at the time of the interview. Demographic variables included age, gender, educational attainment, occupation, and locality. The knowledge domain comprised 17 items, the attitude domain comprised 16 items, and the practice domain comprised 55 items. Full questionnaire items, response options, and scoring procedures are provided in Supplementary Material S1. Higher scores indicated greater knowledge, more prevention-oriented attitudes, and safer behavioral practices.

Field teams followed standardized procedures for participant approach, consent, and questionnaire administration across all venue-time blocks. Data collection sessions were scheduled at different times and days to capture variations in attendance patterns across recruitment sites. Individuals who were unable to provide informed consent or complete the interview were excluded from participation. Show cards and visual prompts were used where appropriate to standardize response options and reduce interviewer-related variability. All interviews were conducted privately after obtaining informed consent. No clinical records were accessed, and no personal identifiers were recorded in the analytical dataset.

Reasons for refusal or partial completion were documented using a standard template. No sampling weights were applied, and all analyses were performed on the unweighted analytic sample.

### 2.3. Minimizing Information Bias

Several measures were implemented to minimize information bias. Interviewers were instructed not to prompt desirable answers and to read each item exactly as written. Where feasible, practice items were framed around recent or routine behaviors to limit recall bias. In addition, field procedures were periodically reviewed to ensure adherence to privacy safeguards and standardized data collection protocols.

### 2.4. Outcomes, Scoring, and Thresholds

Composite KAP scores were the primary outcomes. Scores were calculated by summing item-level responses within each domain according to a predefined scoring scheme. Item coding and domain scoring were prespecified before analysis and are provided in full in Supplementary Material S1. Some items contributed more than one point because selected responses reflected multiple correct or prevention-oriented elements. Accordingly, the 55 practice questions generated 64 coded practice components for scoring. Internal consistency was assessed using Cronbach’s alpha across the combined KAP components, yielding an overall alpha of 0.70, which was interpreted as acceptable for exploratory composite indicators. The composite scores were therefore used as structured domain indicators for descriptive and associational analysis rather than formally validated psychometric scales. The descriptive cutoffs were prespecified before analysis and used for exploratory interpretation based on percentage-of-maximum score categories commonly applied in KAP studies: low/negative scores were defined as <50% of the maximum, moderate/neutral scores were defined as 50% to <75% of the maximum, and high/positive scores were defined as ≥75% of the maximum. These categories were intended to support descriptive interpretation and should not be considered clinically or psychometrically validated thresholds. The knowledge domain comprised 17 items with a maximum score of 21, the attitude domain comprised 16 items with a maximum score of 22, and the practice domain comprised 55 questionnaire items with a maximum score of 64. Higher scores indicated greater knowledge, more prevention-oriented attitudes, and safer behavioral practices.

For descriptive interpretation, knowledge scores were categorized as low (0–10), moderate (11–15), and high (16–21). Attitude scores were categorized as negative (0–10), neutral (11–16), and positive (17–22). Practice scores were categorized as low (0–31), moderate (32–47), and high (48–64). Full scoring details, including item-level coding, are provided in Supplementary Material S1. An additional information-source breadth score was calculated from 0 to 6 to determine the number of reported information-source categories and analyzed separately; this score reflects the breadth rather than the reliability of sources.

### 2.5. Statistical Analysis

Data were analyzed using IBM SPSS Statistics version 23.0 (IBM Corp., Armonk, NY, USA). Continuous variables are presented as means and standard deviations, and categorical variables are presented as counts and percentages. Because the KAP outcomes were summed composite scores across multiple items, they were analyzed as approximately continuous variables for exploratory group comparisons. The distribution of continuous composite scores was assessed prior to analysis. Group differences were examined using independent-samples *t*-tests for variables with two categories and one-way analysis of variance (ANOVA) for variables with more than two categories. ANOVA was used as an omnibus test to assess whether mean scores varied across multi-category predictors; post hoc pairwise comparisons were not performed because these analyses were intended to be descriptive and exploratory. Given the exploratory nature of these analyses, *p*-values were interpreted in conjunction with effect sizes, confidence intervals, and consistency of patterns across domains. Associations between continuous composite scores were assessed using Spearman’s rank correlation coefficient (rho), with two-tailed tests. Separate multivariable linear regression models were fitted for each outcome domain. The dependent variables were the composite knowledge, attitude, and practice scores, as well as the information-source breadth score. Gender was entered as a binary predictor. Age group, educational attainment, and perceived availability of brucellosis treatment were entered as ordered predictors in exploratory trend-based models; this approach assumes an approximately linear trend across ordered categories, and the resulting coefficients were therefore interpreted cautiously. Occupation was included as a numerically coded variable for exploratory model adjustment; because occupational categories are not inherently ordinal, the direction and magnitude of this coefficient were interpreted cautiously. The results are presented as unstandardized beta coefficients with 95% confidence intervals and corresponding *p*-values. Because multiple subgroups and exploratory analyses were conducted, *p*-values were interpreted cautiously, and in conjunction with effect sizes, confidence intervals, and pattern consistency across analyses. Statistical significance was set at *p* < 0.05.

### 2.6. Ethics

The study protocol was reviewed and approved by the Research Ethics Committee of Najran University, Kingdom of Saudi Arabia (Approval No. NU202405-076-020574-047001). Additional approval for data collection was obtained from the Najran Armed Forces Hospital Research Ethics Committee (Approval No. NAFHREC/2024/9). All procedures were conducted in accordance with the Declaration of Helsinki. Written informed consent was obtained from all participants prior to enrollment. To protect participant confidentiality, no names, national identification numbers, or contact details were recorded. Questionnaires were labeled using unique study codes, and electronic data files were password-protected and stored on secure institutional devices accessible only to authorized members of the research team.

## 3. Results

A total of 608 adults were included in the analytic sample. The recruited sample was predominantly male, and women were largely underrepresented. Participants were included across all adult age groups, with the largest proportions among those aged 26–30 years and >41 years. Educational attainment was relatively high, as a substantial proportion of participants were postgraduates, and the sample included both general community members and exposure-relevant occupational groups such as farmers, health professionals, and veterinarians.

### 3.1. Analysis of Brucellosis Information Sources Among the Recruited Sample in Najran City

Family and friends appeared to play a major role as the most frequently reported source of brucellosis-related information, whereas formal channels, including health professionals, media, internet sources, educational institutions, and agricultural or veterinary organizations, were reported much less frequently (Table 1). The information-source breadth score was low, indicating that most participants reported only a limited range of information-source categories.

### 3.2. Overview of Knowledge, Attitude, and Practice Scores Related to Brucellosis in the Recruited Sample

Overall, attitude scores were highest relative to their scale maximum, followed by practice and knowledge scores (Table 2). Most participants had favorable attitudes toward brucellosis prevention. By contrast, knowledge and practice scores were concentrated in the moderate range. This pattern indicates that favorable attitudes were more common than high knowledge level or consistently safer practices. Selected exposure-related and preventive practice behaviors are summarized in Section 3.3.

### 3.3. Frequency of Brucellosis-Related Practices in Najran

Table 3 presents selected brucellosis-related practices grouped around major exposure and prevention pathways. Dairy-related exposure was common, particularly consumption of unpasteurized milk or dairy products (56.6%). Practices for the disposal of abortion material varied; burial was the most frequently reported method (53.3%), while other reported practices included feeding aborted fetuses to dogs (14.5%), dumping them in public areas (11.2%), and discarding them in waterways (4.6%). Biosecurity and personal protective measures were only partially adopted, with approximately half of respondents reporting farm disinfection (51.3%) and use of gloves when handling abortive materials (50.7%). However, mask use (38.8%) and isolation of the aborted animal (32.2%) were less frequently reported. Veterinary-contact and animal-management practices also varied, as 41.4% of the respondents reported contacting a local veterinarian, and 33.6% reported administering medication or vaccination to the aborted animal. Market-related practices were less frequently reported, including slaughtering the aborted animal on the farm (5.3%) and selling aborted animals or raw milk (3.9% each).

### 3.4. Interrelations Among Knowledge, Attitudes, and Practices Concerning Brucellosis

Information-source breadth showed the strongest association with knowledge (rho = 0.561, *p* < 0.0001; Table 4). In contrast, no clear association was found between the breadth of information sources and attitude or practice scores. Attitude showed small positive correlations with both knowledge and practice. The knowledge–practice correlation was weak and inverse. Overall, broader exposure to information sources was mainly associated with higher level of knowledge, whereas correlations involving attitude and practice were small or absent.

### 3.5. Associations of Demographic and Situational Factors with Brucellosis Knowledge, Attitudes, Practices, and Information-Source Breadth

In the two-group comparisons and one-way ANOVA analysis (Table 5), self-reported personal or household experience of brucellosis was associated with higher knowledge scores and slightly more positive attitudes, but not with clearly different practice scores. Compared to men, women had higher attitude scores and practice scores. However, there was no difference in knowledge scores, and women had lower information-source breadth scores. Age effects were observed in all four outcome domains; knowledge increased with age and was highest among participants who were over 40 years old. Practice scores were highest among individuals aged 18–25 years and lowest among those aged 31–40 years. Attitude scores were highest in the 31–40-year age group. Educational attainment was associated with knowledge and practice scores, but not clearly associated with attitude scores. Occupational differences were observed across all outcome domains, with veterinarians showing the highest knowledge and practice scores and non-health professionals showing the lowest practice scores. The perceived availability of brucellosis treatment was mainly associated with practice scores but was not clearly associated with knowledge, attitude, or the breadth of information sources.

### 3.6. Statistical Predictors of Knowledge, Attitude, Practice, and Information-Source Breadth Regarding Brucellosis

In the knowledge model, gender and age showed adjusted associations with knowledge scores; education, occupation, and perceived treatment availability were not associated after adjustment (Table 6). For attitudes, no predictor remained statistically significant after adjusting for covariates. In the adjusted model for practice, gender, age, the coded occupation variable, and perceived treatment availability correlated with practice scores. Variables such as information-source breadth, gender, age, education, and the coded occupation were associated with the breadth of information sources. The direction of the adjusted association between age and knowledge differed from the univariable pattern shown in Table 5.

## 4. Discussion

Brucellosis is an important bacterial zoonosis and remains a re-emerging and often neglected public health concern. Three findings stand out in this venue-based survey of adults in Najran City. First, information on brucellosis was obtained predominantly through informal interpersonal networks rather than formal professional channels. Second, attitudes toward prevention were generally favorable, but safer practices were adopted inconsistently. Third, several exposure-related behaviors remained common, particularly unpasteurized dairy consumption and incomplete biosecurity around animal abortion events. Together, these findings are consistent with the possibility that knowledge-oriented messages alone may be insufficient to improve safer practices. However, because data are cross-sectional, causal relationships cannot be inferred; future intervention studies are needed to determine whether communication and service-delivery strategies improve preventive behaviors.

Information-source findings showed that participants relied mainly on informal interpersonal networks, particularly family and friends, for brucellosis-related information (53.9%), whereas formal sources such as health professionals, the media, and internet sources were reported less frequently (Table 1). This pattern suggests an important communication gap, as reliance on personal networks may shape health knowledge and practices, yet this can also lead to the circulation of incomplete or inaccurate information, as reported in other zoonotic-disease contexts [25,42]. The low proportion of participants reporting health professionals as information sources may reflect limited access to, engagement with, or perceived relevance of formal guidance. Strengthening the role of health professionals remains important, particularly for high-risk groups, as education and training can improve recognition of brucellosis risks and support appropriate preventive responses [43,44]. Similarly, the limited use of media and internet sources suggests that digital and mass-communication platforms may not yet be effectively reaching the target population, despite their potential value for structured health promotion [43,45]. The low reported use of educational institutions and agricultural or veterinary organizations also indicates an opportunity to expand communication on zoonotic diseases through trusted institutional and One Health channels, especially in rural and peri-urban settings [46].

Several structural and cultural factors may help explain the low reliance on formal information sources in this recruited sample. Structurally, access to brucellosis-specific guidance may be limited by fragmented communication between human health, veterinary, agricultural, and community sectors; limited routine counseling at points of care or livestock-contact sites; and the practical cost or availability of preventive resources such as personal protective equipment (PPE), disinfectants, veterinary consultation, and safe disposal support. Culturally, reliance on family and friends may reflect trusted interpersonal networks, household decision-making norms, and the normalization of traditional dairy and animal-handling practices. Intervention designs should therefore combine formal risk communication with trusted community pathways. Practical options include short messages delivered through primary-care and veterinary services, visual instructions at dairy outlets and livestock-contact venues, engagement with community leaders and occupational groups, and gender-responsive education that considers household roles in dairy handling, food preparation, and animal care.

The KAP profile showed moderate knowledge (10.7/21), generally favorable attitudes (19.5/22), and moderate practice scores (36.6/64) (Table 2). This pattern indicates an attitude–practice gap, whereby favorable perceptions toward the prevention of brucellosis do not consistently correspond to safer reported practices. Similar findings have been reported in other zoonotic-disease and brucellosis-related KAP studies, where awareness or positive attitudes alone were insufficient to ensure preventive behavior. This supports the need for interventions that combine education with feasible behavior-change strategies [47]. The moderate knowledge score suggests that gaps remain in public understanding of brucellosis transmission, symptoms, and prevention, consistent with Saudi KAP studies from Qassim and Jazan [20,21]. However, higher awareness was reported in a southern Saudi study involving an older demographic [19]. Understanding transmission routes, symptoms, and preventive measures remains essential for designing effective public health interventions [42]. Although favorable attitudes may provide a useful foundation for engaging with public health [2], the moderate practice score indicates that safer behaviors may still be constrained by insufficient knowledge, limited resources, or practical barriers to implementing recommended measures [30].

The analysis of abortion-related practices and control measures in the recruited sample (Table 3) highlights several modifiable exposure pathways. The results show a high rate of unpasteurized milk consumption at 56.6%, which remained the most frequently reported exposure pathway. Furthermore, multiple unsafe practices related to aborted materials were reported, including disposal in waterways (4.6%) and in streets (11.2%). These practices raise important public health concerns that warrant targeted intervention. Such behaviors may increase the risk of brucellosis transmission, consistent with reports from other agricultural settings. Activities like feeding aborted fetuses to dogs and discarding them in the streets may pose public health risks by potentially spreading brucellosis and other zoonotic diseases [48,49]. Burial of aborted fetuses (53.3%), glove use (50.7%), and mask use (38.8%) suggest partial awareness of the importance of PPE during handling; nevertheless, adherence to recommended practices remained incomplete. The Food and Agriculture Organization (FAO) has noted that wearing gloves during livestock handling is a preventive step to reduce the transmission of brucellosis [50]. It is important to note that burial alone may be insufficient to prevent transmission if not performed correctly, as pathogens can remain in the environment [51,52].

Information-source breadth was moderately associated with knowledge, whereas correlations involving attitude and practice were small or absent (Table 4). A moderate positive correlation was observed between information-source breadth and the knowledge score (rho = 0.561, *p* < 0.0001). This finding is consistent with prior studies, which emphasize the role of broader exposure to information sources in strengthening public health knowledge, with education demonstrating a positive association with awareness and knowledge levels [39,53,54]. The observed positive correlations between attitude and both knowledge (rho = 0.158, *p* < 0.0001) and practice scores (rho = 0.157, *p* < 0.0001) were small but statistically significant, suggesting that more favorable attitudes may modestly support both knowledge and practice outcomes. Consistent with previous studies, we did not observe a significant relationship between information-source breadth and either attitude or practice scores [14,39]. Conversely, knowledge and practice scores showed a weak inverse correlation. This finding should be interpreted cautiously rather than inferring that knowledge limits safer practice. Alternative explanations include residual confounding, differences in occupational exposure, measurement limitations, or the possibility that participants with greater knowledge were more likely to engage in livestock-related tasks where risk practices are relevant. Therefore, the finding should be viewed as hypothesis-generating and supportive of behavior-focused intervention research rather than evidence of a causal knowledge–practice relationship.

Demographic and occupational patterns suggested variations in KAPs and the information-source breadth across participant groups; however, these findings should be interpreted as exploratory due to the venue-based sampling design, multiple comparisons, and possible residual confounding. Participants who reported personal or household experience of brucellosis had a higher level of knowledge than those without such experience, consistent with previous studies suggesting that firsthand experience with a health condition may influence understanding [55,56].

Gender was also associated with differences in brucellosis-related perceptions and practices, with males demonstrating less favorable attitudes than females (Table 5). This pattern is consistent with the fact that more cautious attitudes towards health are most often observed among females in comparable contexts [57]. Furthermore, gender emerged as a significant predictor of practices (Table 6), suggesting that practice patterns differed by gender. This finding contrasts with other studies where gender differences were negligible, implying that interventions might need to be explicitly tailored to gender in Najran. These findings are partly consistent with previous research, which reported higher levels of brucellosis awareness and knowledge in some male and professionally exposed groups, including healthcare workers and individuals with higher education levels in Jordan [11]. The association between gender and information-source breadth suggests that access to brucellosis-related information may differ by gender in this recruited sample. Men in this sample appeared to report broader access to information sources than women, which aligns with prior research suggesting that the transfer of brucellosis-related knowledge may vary by gender, with veterinarians frequently providing more details to men within the community [57]. Another study in Taif City, Saudi Arabia, found that 30.6% of males demonstrated satisfactory knowledge, which is significantly higher than 14.9% of females (*p* < 0.001) [58].

Age-related differences in knowledge of brucellosis were noteworthy, with individuals aged 18–25 years demonstrating lower knowledge scores. This finding supports the need for age-targeted educational interventions. These align with other areas of public health research that stress the importance of age-appropriate outreach [2]. Conversely, the adjusted regression model showed a positive association between age and practice scores (*β* = 1.75, Table 6). This pattern aligns with observations from the Qassim region and other parts of Saudi Arabia, where awareness levels were markedly lower among younger populations [21]. Age was also negatively associated with information-source breadth (*β* = −0.562, Table 6), which indicates differences in exposure to or engagement with relevant health information across age groups. This observation emphasizes the importance of specifically tailored educational strategies to younger populations. Past studies in various areas of the Kingdom of Saudi Arabia have shown that older people understand brucellosis better, supporting the need for age-appropriate awareness campaigns and educational programs [19,20].

Occupation was significantly associated with brucellosis-related knowledge and practices. Farmers scored higher in knowledge assessments (Table 5), likely due to their frequent interactions with livestock, which is a known risk factor [59]. These patterns are consistent with evidence that occupational exposure can shape zoonosis-related health behaviors [60]. In this sample, participants in non-health professions had the lowest practice scores. Occupation remained statistically associated with practice scores in the adjusted model; however, because occupation was coded for model inclusion rather than modeled as a full set of dummy categories, the direction and magnitude of this coefficient should be interpreted cautiously and considered exploratory. Occupation was also independently associated with information-source breadth (*β* = 0.151), suggesting that professional context may influence access to brucellosis-related information. This finding suggests that farmers and veterinarians may function as influential community channels for brucellosis-related information. Nevertheless, the lower practice scores among non-health professionals emphasize the need for targeted, occupation-specific educational and behavioral interventions to strengthen brucellosis prevention, risk reduction, and appropriate care-seeking [60,61]. These findings align with evidence emphasizing the dual importance of protecting occupationally exposed individuals and maintaining food safety across the livestock and dairy value chains. In this context, delayed care-seeking behavior and low clinical suspicion—primarily due to limited awareness and the normalization of risk—may prolong the diagnostic interval and hinder the prompt initiation of appropriate treatment. Such delays can worsen individual morbidity and may prolong exposure risks in household or occupational settings [60,61,62].

Several factors may explain why the differences observed across occupational groups were smaller than expected, including score compression, shared structural constraints, and task-level heterogeneity. Three main explanations are plausible. First, score compression—such as ceiling effects observed in attitude scales—diminished the sensitivity to differences between groups; attitudes were frequently clustered near the upper end, thereby complicating the detection of genuine differences. Second, structural and normative elements—such as the accessibility and cost of PPE and disinfectants, prompt access to veterinary advice, market incentives for raw dairy, and enduring familial and workplace norms—may diminish occupational distinctions, resulting in the well-documented attitude–practice gap reported in brucellosis KAP studies across various contexts [13,14,30]. Third, our composite practice score, which encompasses a range of tasks (e.g., handling abortion materials, milking, marketing, and carcass disposal), has the potential to obscure meaningful task-specific differences within the same occupational groups. Research from Ethiopia and Sri Lanka demonstrates considerable within-occupation variation in behaviors such as glove usage during parturition, disinfection, and veterinary consultation, suggesting that behaviors may cluster more strongly by contextual constraints than by job title alone [13,14].

Because attitudes were generally favorable while practices remained weaker, interventions should combine risk communication with practical behavior-change supports. Examples include visual instructions at dairy points of sale and livestock-contact sites, short mobile reminders during high-risk periods, easier access to PPE and disinfectants, veterinary prompts for timely advice, and gender-responsive community delivery approaches that consider household roles in dairy handling, food preparation, and animal care [12,63,64]. These strategies are consistent with One Health principles because they require coordinated action across human health, veterinary, food-system, and community sectors [64]. Applying the capability–opportunity–motivation framework may also support sustainable changes in behavior beyond the provision of information alone [65]. Because unpasteurized dairy consumption was the most frequently reported exposure-related behavior in this study, dairy-safety interventions should address locally relevant livestock sources and informal milk-distribution practices. In settings where camel, goat, sheep, or cow milk may be consumed fresh or informally distributed, messages should emphasize boiling, pasteurization, and avoiding raw milk from animals with suspected infections or that have undergone recent abortion [12,63,66].

The adjusted models provide exploratory evidence that demographic, occupational, and service-access factors may influence KAPs and information-source breadth. Gender remained independently associated with knowledge and practice scores, illustrating an awareness–behavior dissociation that has been reported in other Saudi KAP studies, where male awareness typically exceeds measures put into practice. As such, daily protective behaviors display gender-based variability [20,21,67]. Differences between univariable and adjusted patterns, particularly for age and knowledge, may reflect confounding, suppression effects, or correlation among age, education, and occupation [68]. Similarly, occupation-related coefficients should be interpreted cautiously because these categories reflect heterogeneous tasks and exposure contexts. However, work environments and professional networks remain important for biosecurity practices and communication channels [14,67]. The association between perceived treatment availability and practice may reflect the role of service access and other enabling factors in supporting preventive behavior, consistent with health-services models [69,70]. The more complex pattern for information-source breadth may also reflect channel substitution across occupational networks and age-related differences in digital information-seeking [71]. Overall, these adjusted findings support targeted risk communication and practical service enablers. However, they should be viewed as hypothesis-generating rather than causal [67,69,70].

Educational associations in this sample were mixed. Knowledge tended to be greater at higher educational levels, whereas attitudes did not differ significantly across education categories, and practice scores did not increase monotonically with education. Likewise, a study conducted in the Kingdom of Saudi Arabia among livestock farmers and meat handlers indicated that increased education was associated with safer practices and greater concern about brucellosis [67]. Several studies included in the meta-analysis have shown a positive correlation between awareness and knowledge levels and educational attainment [39,54]. Educational level was significantly associated with information-source breadth in the adjusted analysis (Table 6). Because education was entered as a coded ordinal predictor and subgroup means were not strictly monotonic, the direction of this association should be interpreted cautiously. Overall, the findings suggest heterogeneity in how educational groups access brucellosis-related information rather than a simple linear gradient. This observation is consistent with broader health education research, which demonstrates that higher educational attainment generally enhances access to information and strengthens the ability to understand and retain health-related knowledge [72].

Brucellosis also has important economic implications for households and health systems. Previous research has shown substantial social benefits from livestock vaccination and improved diagnostic approaches. Integrating economic considerations into prevention messaging may be justified, for example, by combining straightforward dairy-safety guidance with practical indicators such as the number of workdays saved or reduced losses associated with unsafe dairy practices. However, economic awareness and household financial losses were not directly assessed in this study. Accordingly, these considerations should be interpreted as policy-relevant context rather than measured findings from the present dataset. Future studies could include a brief economic-awareness module to evaluate whether linking prevention messages to household and livestock losses improves protective practices [73].

## 5. Conclusions

Among adults recruited through venue-based time–location sampling in Najran City, we observed moderate knowledge, generally favorable attitudes, important gaps in preventive practices, and substantial reliance on informal information channels. These patterns highlight opportunities to strengthen risk communication through formal health and veterinary services and to align human, animal, and food-chain actions within a One Health framework. Practical implementation could include joint health–veterinary messages on animal abortion management, visible safe-dairy guidance at markets and dairy outlets, referral prompts for suspected animal or human cases, and occupational training for farmers, slaughterhouse workers, dairy vendors, and other groups exposed to livestock. However, the venue-based design and demographic imbalance in this study should be taken into consideration when interpreting the findings and prioritizing future probability-based studies. Within these constraints, the study identifies actionable communication and service-delivery entry points that can be tested and evaluated through future implementation and monitoring efforts. These findings can inform locally adapted brucellosis prevention planning in Najran and in comparable settings by supporting evidence-based messaging at dairy points of sale, workplace- and community-based training for high-exposure groups, and measurable One Health indicators for monitoring and evaluation. Future research should evaluate whether these interventions improve practices over time using longitudinal designs and, where feasible, integrate serological surveillance or routine reporting data to strengthen causal inference and population-level interpretation.

## 6. Limitations

This study should be interpreted in light of several limitations. First, recruitment relied on a venue-based time–location approach rather than a household probability sampling framework. The final sample reflected differential participation across major demographic strata. It included an underrepresentation of women and relatively high participation of respondents with advanced educational attainment. This pattern may partly reflect the recruitment of participants through institutional hubs, favoring institutionally affiliated individuals. In the absence of sampling weights, the study is best interpreted as providing descriptive and associational insights within the recruited sample, and generalization to the wider adult population should therefore be made with caution. Key patterns warrant confirmation in probability-based surveys before population-level inferences can be made for Najran City. Second, although the KAP instrument, scoring scheme, and descriptive cutoffs were adapted from existing brucellosis KAP tools and refined through local expert review, the instrument was not formally psychometrically validated in the local study population. However, because knowledge, attitude, and practice represent conceptually distinct domains, this overall reliability estimate should be interpreted cautiously, and the domain scores should be viewed as structured composite indicators rather than fully validated psychometric scales. Third, some high-risk occupational groups (e.g., veterinarians and slaughterhouse workers) had relatively small sample sizes, reducing the precision of subgroup comparisons. Fourth, exposures and outcomes were self-reported within a cross-sectional design, introducing recall and social desirability bias while precluding causal inference; consequently, unsafe practices may have been underreported, and the magnitude of the attitude–practice gap may have been underestimated or mischaracterized. The role of volunteer or community organizations in brucellosis prevention was not directly assessed in this survey and should be examined in future implementation-focused studies. Finally, although multivariable models were adjusted for key covariates, residual confounding remained possible, and some associations may reflect chance findings arising from multiple comparisons; these results should be interpreted cautiously. Future studies should strengthen inference through probability-based household sampling and mixed-methods designs and, where feasible, incorporate longitudinal follow-up and serological or registry-based data to validate reported exposure histories and outcomes.

## Figures and Tables

**Table 1:** Information-source breadth on brucellosis reported by the recruited sample.

Information-Source Breadth	*n*	%
**Family or friends**		
No	280	46.1
Yes	328	53.9
**Health professionals**		
No	560	92.1
Yes	48	7.9
**Media**		
No	580	95.4
Yes	28	4.6
**Internet**		
No	588	96.7
Yes	20	3.3
**Educational institutions**		
No	596	98.0
Yes	12	2.0
**Agricultural and veterinary organizations**		
No	600	98.7
Yes	8	1.3

The information-source breadth score summed six yes/no items about whether participants reported obtaining brucellosis information from each source, ranging from 0 (none) to 6 (all). Information-source breadth ranged from 0 to 6, with a mean ± SD of 0.7 ± 1.0.

**Table 2:** Knowledge, attitude, and practice scores regarding brucellosis (N = 608).

Variables	Number of Questions	Total Score	Total Score * (Mean ± SD)	Level (%)		
				Low	Moderate	High
Knowledge	17	21	10.7 ± 2.8	13.8	75.7	10.5
Attitude	16	22	19.5 ± 4.3	0.7	12.5	86.8
Practice	55	64	36.6 ± 6.5	0	63.2	36.8

* Maximum scores were predefined for each domain. Higher scores reflect better knowledge, more prevention-oriented attitudes, and safer practices.

**Table 3:** Selected brucellosis-related exposure behaviors and preventive practices in the recruited sample from Najran City.

Practices	*n*	%
Fed aborted fetuses to dogs	88	14.5
Administered medication or vaccination to an aborted animal	204	33.6
Disposed of aborted fetuses in water channels	28	4.6
Discarded aborted fetuses in the streets	68	11.2
Sold aborted animals in the market	24	3.9
Slaughtered the aborted animal on the farm	32	5.3
Contacted a local veterinarian	252	41.4
Sold the aborted animal to the butcher	24	3.9
Separated the aborted animal from the other animals	196	32.2
Burned an aborted fetus	40	6.6
Wore protective gloves when disposing of an aborted fetus	308	50.7
Disinfected the farm	312	51.3
Wore a protective mask when disposing of an aborted fetus	236	38.8
Consumed unpasteurized milk or dairy products	344	56.6
Sold raw milk	24	3.9
Buried an aborted fetus	324	53.3

**Table 4:** Correlations among knowledge, attitude, and practice scores related to brucellosis in the recruited sample from Najran City.

Variables	Rho *	*p*-Value
Information-source breadth and attitude score	0.004	0.928
Information-source breadth and knowledge score	0.561	<0.0001
Information-source breadth and practice score	−0.055	0.172
Attitude score and knowledge score	0.158	<0.0001
Attitude score and practice score	0.157	<0.0001
Knowledge score and practice score	−0.123	0.002

* Spearman’s rank correlation coefficient (rho); *p*-values < 0.05 were considered statistically significant.

**Table 5:** Associations of demographic and situational factors with knowledge, attitude, practice scores, and information-source breadth for brucellosis in the recruited sample from Najran City (N = 608).

Factors	Knowledge			Attitude			Practice			Information-Source Breadth		
	Mean ± SD	t/F	*p*	Mean ± SD	t/F	*p*	Mean ± SD	t/F	*p*	Mean ± SD	t/F	*p*
Do you or any member of your household have personal experience related to brucellosis?												
Yes (27%)	12.8 ± 1.9	−12.3	<0.0001	20.1 ± 3.7	−2.3	0.02	35.9 ± 5.4	1.7	0.08	1.05 ± 0.9	−4.7	<0.0001
No (73%)	9.9 ± 2.7			19.2 ± 4.5			36.9 ± 6.8			0.6 ± 0.9		
Gender												
Male	10.7 ± 2.8	1.1	0.2	19.3 ± 4.3	−2	0.04	36.5 ± 6.3	−2	0.04	0.78 ± 1.0	3.7	<0.0001
Female	10.3 ± 2.3			20.6 ± 4.6			38.3 ± 7.5			0.27 ± 0.5		
Age												
18–25	7.5 ± 0.5	37.6	<0.0001	18.0 ± 4.2	3.2	0.02	39.0 ± 5.3	19.8	<0.0001	0.20 ± 0.6	4.5	0.004
26–30	9.5 ± 2.9			18.9 ± 4.0			38.9 ± 6.5			0.60 ± 1.0		
31–40	11.5 ± 2.6			20.2 ± 4.0			35.0 ± 5.6			0.92 ± 1.0		
>41	11.7 ± 2.2			19.7 ± 4.8			35.0 ± 6.0			0.81 ± 0.9		
Education												
Secondary education	10.4 ± 3.1	20.5	<0.0001	19.0 ± 4.1	2.464	0.06	37.6 ± 6.0	17.9	<0.0001	0.65 ± 1.0	8.2	<0.0001
Bachelor’s degree	9.0 ± 2.4			19.6 ± 3.3			40.0 ± 6.4			0.35 ± 0.4		
Master’s degree	11.3 ± 2.4			19.5 ± 4.8			35.1 ± 6.2			0.92 ± 1.0		
PhD	11.6 ±2.9			21.1 ± 3.7			34.6 ± 6.3			0.67 ± 0.6		
Occupation												
Farmer	12.7 ± 4.6	32.6	<0.0001	20.1 ± 4.1	3.779	0.005	36.5 ± 5.2	21.3	<0.0001	1.5 ± 2.1	12.6	<0.0001
Student	9.1 ± 2.3			18.5 ± 4.3			39.6 ± 6.5			0.4 ± 0.7		
Non-health professional	11.4 ± 2.1			19.6 ± 4.5			34.2 ± 5.2			0.8 ± 0.8		
Health professional	10.9 ± 2.6			20.3 ± 4.0			36.3 ± 7.0			0.6 ± 0.8		
Veterinarian	13.3 ± 1.3			20.3 ± 2.9			40.6 ± 3.8			0.6 ± 0.4		
Perceived availability of brucellosis treatment in Najran												
Very dissatisfied (8.6%)	11.1 ± 2.0	1	0.3	20.5 ± 4.8	1.6	0.1	34.1 ± 5.0	24.0	<0.0001	0.6 ± 0.4	0.5	0.7
Not satisfied (7.2%)	11.1 ± 2.7			18.9 ± 5.9			33.0 ± 6.7			0.6 ± 0.4		
Neutral (29.6%)	10.4 ± 3.5			19.7 ± 4.2			35.0 ± 6.3			0.7 ± 1.3		
Satisfied (34.2%)	10.8 ± 2.4			19.0 ± 4.0			37.0 ± 5.8			0.7 ± 0.8		
Very satisfied (20.4%)	10.5 ± 2.6			19.6 ± 4.1			40.7 ± 6.0			0.6 ± 1.1		

Values are presented as mean ± SD. Two-group comparisons were performed using independent-samples *t*-tests, whereas multi-category comparisons were performed using one-way ANOVA (F). Two-sided *p*-values were used, and statistical significance was set at *p* < 0.05. Score ranges were knowledge 0–21, attitude 0–22, practice 0–64, and information-source breadth 0–6.

**Table 6:** Predictors of knowledge, attitude, practice scores, and information-source breadth regarding brucellosis in the recruited sample from Najran City (N = 608).

Predictors	Knowledge			Attitude			Practice			Information-Source Breadth		
	*β* *	95% CI	*p*	*β*	95% CI	*p*	*β*	95% CI	*p*	*β*	95% CI	*p*
Constant	7.4	6.2 to 8.6	<0.0001	16.7	14.8 to 18.7	<0.0001	37.74	35.0 to 40.4	<0.0001	0.731	0.28 to 1.17	0.001
Gender	1.00	0.80 to 1.30	<0.0001	0.27	−0.13 to 0.60	0.19	−1.23	−1.78 to −0.67	<0.0001	0.129	0.03 to 0.22	0.007
Age	−0.82	−1.50 to −0.08	0.02	1.00	−0.19 to 2.20	0.10	1.75	0.11 to 3.39	0.036	−0.562	−0.83 to −0.28	<0.0001
Education	0.04	−0.21 to 0.30	0.73	0.24	−0.18 to 0.66	0.26	−0.23	−0.81 to 0.33	0.411	−0.142	−0.23 to −0.04	0.004
Occupation	0.20	−0.03 to 0.45	0.09	0.14	−0.25 to 0.50	0.47	−0.83	−1.38 to −0.29	0.003	0.151	0.06 to 0.24	0.001
Perceived treatment availability	0.08	−0.10 to 0.20	0.39	−0.15	−0.46 to 0.10	0.32	1.62	1.20 to 2.00	< 0.0001	0.055	−0.01 to 0.12	0.12

* Beta coefficients (*β*) were derived from multivariable linear regression models. Values are presented as *β*, 95% confidence intervals (CIs), and two-sided *p*-values. Statistical significance was set at *p* < 0.05.

## Data Availability

The data will be made available on reasonable request.

## Supplementary Materials

The following supporting information can be downloaded at: https://www.mdpi.com/article/10.3390/tropicalmed11060149/s1. Supplementary Material S1 comprises the study questionnaire, encompassing all items, response options, and the scoring protocol. Supplementary Material S2: Illustrative One Health implementation considerations and candidate monitoring indicators.

## Abbreviations

CI	Confidence interval
rho	Correlation coefficient
FAO	Food and Agriculture Organization
KAPs	Knowledge, attitudes, and practices
MEWA	Ministry of Environment, Water, and Agriculture (Kingdom of Saudi Arabia)
NAFH	Najran Armed Forces Hospital
SPSS	IBM SPSS Statistics
SD	Standard deviation
t/F	t: *t*-statistic from a *t*-test; F: F-statistic from an ANOVA (analysis of variance) test
PPE	Personal protective equipment
