# Supplementary material for: Knowledge, Attitudes, Practices, and Information Pathways Related to Brucellosis Among Adults in Najran City, Saudi Arabia: A Stratified Time–Location Cross-Sectional Study
Source: Trop Med Infect Dis. 2026 May 29;11(6):149. doi: 10.3390/tropicalmed11060149 (PMC13307892; doi:10.3390/tropicalmed11060149)
Supplement: Supplementary file 1 [file tropicalmed-11-00149-s001.zip › Supplementary S1.pdf]

Supplementary Material S1: Comprises the study questionnaire, encompassing all items, response options, and the scoring protocol.

A) Demographic Characteristics:

1. What is your age?
1) 18-25
2) 26-30
3) 31-40
4) more than 41

2. What is your gender?
1) Male
2) Female

3. What is your highest level of education?
1) Illiterate
2) Secondary Education
3) Bachelor's degree
4) Master's degree
5) PhD

4. What is your occupation?
1) Farmer/livestock-contact worker/butcher
2) Student
3) Non-health professional
4) Health professional
5) Veterinarian

B) Knowledge Questions

The knowledge domain comprised **17** items, with a maximum possible score of **21**. Higher scores indicated greater brucellosis-related knowledge. To evaluate awareness and understanding, the questionnaire included the following questions:

- **General Awareness**:

1. (**K1**) Have you ever heard of brucellosis?
1) No (score :0)
2) Yes (score:1)

2. (**K2**) Have you or anyone in your family ever had brucellosis?
1) No (score :0)
2) Yes (score:1)

3. (**K3**) Have you heard of brucellosis as an animal disease?
1) No (score :0)
2) Yes (score:1)

4. (**K4**) Have you heard of brucellosis as a human disease?
1) No (score :0)

2) Yes (score:1)

5. (**K5-10**) Where did you get your information about brucellosis from? (Select all that apply) *

1) Family or friends (**K5**, score: no=0, yes=1)
2) Health professionals (**K6**, score: no=0, yes=1)
3) Media (**K7**, score: no=0, yes=1)
4) Internet (**K8**, score: no=0, yes=1)
5) Educational institutions (**K9**, score: no=0, yes=1)
6) Agricultural or veterinary organizations (**K10**, score: no=0, yes=1)
7) Other (please specify): _______

6. (**K11**) Which animals are susceptible to brucellosis? (Select all that apply) *

1) Avian (score :0)
2) Some of the domestic (cows, sheep, goats) (score:1)
3) Domestic livestock and other susceptible animals, including cattle, sheep, goats, camels, and dogs where relevant (score :2)

- **Human Infection:**

7. (**K12**) What are the symptoms of brucellosis in humans? (Select all that apply) *

1) No (score :0)
2) Partial information (Fever, Sweating, Fatigue, Headache) (score:1)
3) Yes (Fever, Sweating, Fatigue, Joint pain, Muscle pain, Headache) (score:2)

8. (**K13**) Is there a vaccine available for animals to prevent brucellosis?

1) No (score:0)
2) Not sure(score:1)
3) Yes (score:2)

9. (**K14**) How is brucellosis transmitted between animals?

1) Direct contact with infected animals or contaminated food or water (score: True=1)
2) Inhalation of airborne agents (score: False=0)
3) Other (please specify): _______

10. (**K15**) Are you aware of the ways in which brucellosis is transmitted from animals to humans? (Select all that apply)

1) Contact with infected people (false, score=0)
2) Contact with infected animals or their by-products (Consumption of unpasteurized milk (raw milk) - Consumption of other unpasteurized dairy products - Contact with aborted fetus or placental membrane) (true, score=1)

- **Previous Infections**:

11. (**K16**) Have any of your livestock (cows, sheep, goats) ever been infected with brucellosis?

1) No (score:0)
2) Not sure (score:1)
3) Yes (score:2)

12. (**K17**) What clinical signs do you know that can be observed in animals infected with brucellosis? (Select all
that apply)

1) Nervous symptoms (score: false=0)

2) Abortion and infertility (score: true=1)
3) Respiratory symptoms (score: false=0)
4) other

C) Attitudes and dairy-related risk-perception items toward brucellosis:

This section assessed perceived risk, prevention-oriented attitudes, and dairy-related risk perceptions. The attitude section comprised 16 points assessed on a scale, with a maximum possible score of 22. The questions consisted of:

1. (**A1**) Would you like more information about brucellosis?
   1) No (score: 0)
   2) Yes (score: 1)

2. (**A2-7**) What is your preferred source for information about brucellosis? (Select all that apply)? *
   1) A2: Family or friends (A2, score: no=0, yes=1)
   2) A3: Health professionals (A3, score: no=0, yes=1)
   3) A4: Media (A4, score: no=0, yes=1)
   4) A5: Internet (A5, score: no=0, yes=1)
   5) A6 Educational institutions (A6, score: no=0, yes=1)
   6) A7: Agricultural or veterinary organizations (A7, score: no=0, yes=1)
   7) Other (please specify): ________

- **Perceived Risk**

3. (**A8**) Do you think any of your family members are at risk of catching brucellosis?
   1) No (score :0)
   2) Not sure (score:1)
   3) Yes (score:2)

4. (**A9-11**) Who do you believe is most vulnerable to contracting brucellosis in your household? (Select all that apply)? *
   1) Elderly people (**A9**, score: no=0, yes=1)
   2) Children (**A10**, score: no=0, yes=1)
   3) Pregnant women (**A11**, score: no=0, yes=1)
   4) Other (please specify):_______

- **Milk Sales and Consumption**

5. (**A12**) Do you sell unpasteurized milk or dairy products directly to consumers?
   1. No (score :1)
   2. Yes (score:0)

6. (**A13**) Do you boil raw milk before making yogurt, cheese, cream, or butter?
   1. Never (score:0)
   2. Sometimes (score:1)
   3. Regularly (score:2)

7. (**A14**) How often do you consume unpasteurized milk or dairy products?
   1. Daily (score:0)
   2. Weekly (score:1)

3. Monthly (score:2)
4. Rarely (score:3)
5. Never (score:4)

8. (**A15**) Does your family consume unpasteurized milk or dairy products?
1. No (score:1)
2. Yes (score:0)

9. (**A16**) Do you think any abortion in cows, sheep, or camels is caused by brucellosis?
1. No (score:0)
2. I have no idea (score:1)
3. Yes (score:2)

D) Frequency of PRACTICE regarding Brucellosis:

This section discusses practices concerning the consumption of raw milk, arrangements for living with animals, hygiene protocols during milking and livestock management, animal health, and traditional medicinal practices. The practice domain comprised 55 questionnaire items, generating 64 coded practice components, with a maximum possible score of 64. The questions included:

1. (**P1**) If you have livestock in your home or your family's home, do you wash your hands before and after milking, for example, camels, cows, or sheep?
1. Never (score:0)
2. Sometimes (score:1)
3. Always (score:2)

2. (**P2**) Who usually helps during animal parturition in your home?
1. Family Members (score:0)
2. Hired Help (score:1)
3. Veterinarian (score:2)

3. (**P3**) Is the place disinfected after birth?
1. No (score:0)
2. Yes (score:1)

4. (**P4**) Are placental membranes and aborted fetuses disposed of by burying them?
1. No (score:0)
2. Yes (score:1)

5. (**P5**) Do you or your assistant wear protective clothing when handling animals (cows, sheep, camels) that had a miscarriage or when handling abortive materials?
1. No (score:0)
2. Yes (score:1)

6. (**P6**) Do you clean up the manure regularly?
1. No (score:0)
2. Yes (score:1)

7. (**P7**) Do you clean the feeding and water troughs regularly?
1. No (score:0)
2. Yes (score:1)

8. (**P8**) Do you store manure piles for more than 6 months?
1. No (score:1)
2. Yes (score:0)

9. (**P9**) Can your animals access manure piles?
1. Yes (score:0)
2. No (score:1)

10. (**P10**) Are animals sent for shared grazing?
1. Yes (score:0)
2. No (score:1)

11. (**P11-14**) Do you consult a veterinarian regarding animal health issues? (Select all that apply) *
1. Veterinarian (**P11**, score: no=0, yes=1)
2. Agricultural extension officer (**P12**, score: no=0, yes=1)
3. Other farmers (**P13**, score: no=0, yes=1)
4. Family members (**P14**, score: no=0, yes=1)
5. Other (please specify): ________

12. (**P15-19**) What do you usually do with aborted animal fetuses? (Select all that apply) *
1. Bury them (**P15**, score: no=0, yes=1)
2. Burn them (**P16**, score: no=0, yes=1)
3. Dispose of them in water canals (**P17**, score: no=1, yes=0)
4. Dispose of them in the streets (**P18**, score: no=1, yes=0)
5. Feed them to dogs (**P19**, score: no=1, yes=0)
6. Other (please specify): ________

13. (**P20**) Does the birthing area open and share with other animals?
1. Yes (score:0)
2. No (score:1)

- **Management and Protective Measures**

14. (**P21-28**) What actions do most livestock owners take when an animal with brucellosis is detected or suspected? (Select all that apply) *
1. Slaughter the suspected or infected animal on the farm (**P21**, score: no=1, yes=0)
2. Sell the suspected or infected animal in the market (**P22**, score: no=1, yes=0)
3. Give medications to the suspected or infected animal (**P23**, score: no=1, yes=0)
4. Call the local veterinarian (**P24**, score: no=0, yes=1)
5. Separate the suspected or infected animal from others (**P25**, score: no=0, yes=1)
6. Remove the identified animal from the herd through sale (**P26**, score: no=1, yes=0)
7. Slaughter animals when they suspect brucellosis (**P27**, score: no=1, yes=0)
8. Vaccinate the suspected or infected animal (**P28**, score: no=0, yes=1)
9. Other (please specify): _______

15. (**P29-32**) What practices are followed when an animal gives birth? (Select all that apply) *
1. Assist with parturition (**P29**, score: no=1, yes=0)
2. Wear protective gloves when helping with parturition (**P30**, score: no=0, yes=1)
3. Wear a protective mask when helping with parturition (**P31**, score: no=0, yes=1)
4. Disinfect the birthing area (**P32**, score: no=0, yes=1)
5. Other (please specify): ________

16. (P33-45) What practices are followed when an animal aborts? (Select all that apply) *

1. Feed aborted fetus to dogs (**P33**, score: no=1, yes=0)
2. Administer medication or vaccination to the aborted animal(**P34**, score: no=1, yes=0)
3. Dispose of aborted fetus in water canals (**P35**, score: no=1, yes=0)
4. Dispose of aborted fetus in the streets (**P36**, score: no=1, yes=0)
5. Sell aborted animal in the market (**P37**, score: no=1, yes=0)
6. Slaughter aborted animal on the farm (**P38**, score: no=1, yes=0)
7. Call the local veterinarian (**P39**, score: no=0, yes=1)
8. Sell aborted animal to the butcher (**P40**, score: no=1, yes=0)
9. Separate aborted animal from other animals (**P41**, score: no=0, yes=1)
10. Burn aborted fetus (**P42**, score: no=0, yes=1)
11. Bury the aborted fetus (**P43**, score: no=0, yes=1)
12. Wear protective gloves when disposing of an aborted fetus (**P44**, score: no=0, yes=1)
13. Wear a protective mask when disposing of an aborted fetus (**P45**, score: no=0, yes=1)
14. Other (please specify): ________

17. (P46) Do you cover hand wounds when in contact with animals?

1. Never (score:0)
2. Sometimes (score:1)
3. Always (score:2)

- **Health Assurance Practices**

18. (P47) Do you take any steps to ensure the health of the new livestock you purchase?

1. No (score:0)
2. Yes (score:1)

19. (P48) Should you contact a veterinarian if you suspect brucellosis in animals on your farm?

1. No (score:0)
1. Yes (score:1)

20. (P49-52) What actions do you take if your cattle show signs of illness? (Select all that apply) *

1. Administer medication (**P49**, score: no=0, yes=1)
2. Isolate the sick animal (**P50**, score: no=0, yes=1)
3. Use traditional treatments (**P51**, score: no=1, yes=0)
4. Consult other farmers (**P52**, score: no=1, yes=0)
5. Other (please specify): ________

21. (P53) If you are diagnosed with brucellosis, where will you seek treatment?

1. Traditional healer (Score:0)
2. Private doctor (Score:1)
3. Local clinic (Score:2)
4. Hospital (Score:3)
5. Other (please specify): ________

22. (P54) How satisfied are you with the availability of brucellosis treatment in your area?

1. Very dissatisfied (Score:0)
2. Not satisfied (Score:1)
3. Neutral (Score:2)
4. Satisfied (Score:3)
5. Very satisfied (Score:4)

- Beliefs and Practices

23. (**P55**) Do you sell raw milk to neighbors?

1. Yes (Score:0)
2. I have no idea (Score:1)
3. No (Score:2)
