# Supplementary material for: Knowledge, Attitudes, Practices, and Information Pathways Related to Brucellosis Among Adults in Najran City, Saudi Arabia: A Stratified Time–Location Cross-Sectional Study
Source: Trop Med Infect Dis. 2026 May 29;11(6):149. doi: 10.3390/tropicalmed11060149 (PMC13307892; doi:10.3390/tropicalmed11060149)
Supplement: Supplementary file 1 [file tropicalmed-11-00149-s001.zip › Supplementary S2.pdf]

Supplementary Material S2: Illustrative One Health implementation considerations and candidate monitoring indicators.

This supplementary framework presents implementation-oriented considerations informed by the observed KAP gaps in the current survey. The proposed actions and indicators are illustrative and should be adapted to local mandates, feasibility, and existing data systems. The survey did not directly evaluate policy performance, program implementation, or intervention effectiveness.

i. **Support cross-sector One Health coordination**
A stronger One Health approach to brucellosis control would benefit from closer coordination across human health, animal health, and community-facing sectors. Practical steps may include reviewing existing brucellosis-related activities, establishing a structured mechanism for joint planning, and evaluating current surveillance and reporting systems.

ii. **Integrate KAP-informed education into routine services**
Primary healthcare, veterinary services, and community nursing outreach can work together to include brucellosis risk assessments and customized health strategies, improving community health and safety education. Special attention should be given to younger individuals, women, and non-health workers, as the study identified subgroup differences in KAP scores and information-source access. Given observed gender differences, gender-responsive delivery may improve uptake—particularly in rural/peri-urban contexts. Examples include leveraging female community health workers and women-focused settings (e.g., antenatal clinics, family medicine visits, female campuses, and women's community gatherings) to deliver household-centered modules on dairy handling and home-based animal care, while engaging household decision-makers who influence purchasing and food preparation.

iii. **Align animal health programs with public health messaging**
Explicit integration with harmonized public health messages could improve existing livestock vaccination activities, abortion reporting, and safe disposal protocols. Veterinarians, municipal personnel, and healthcare workers are recommended to provide consistent, evidence-based guidance on the prevention and control of brucellosis.

iv. **Enhance integrated surveillance and feedback mechanisms**
Cross-sector information sharing between human-health and animal-health services is a core One Health principle and may strengthen early detection and coordinated response to zoonotic threats. Where feasible, local stakeholders could consider routine exchange of aggregated information on suspected animal abortions, confirmed animal cases, suspected human cases, and selected exposure indicators, accompanied by periodic joint review (Communicable Diseases - Malta Fever.

v. **Utilize digital and community-based platforms for One Health communication**
Digital tools and community channels, including farmer associations, schools, mosques, and community-based organizations or local civic groups, where available, should be utilized to disseminate standardized and culturally sensitive One Health messages regarding brucellosis. Evidence-based content should be uniform across various sectors and crafted to foster sustainable behavioral modifications in both human and animal health practices. Operationally, digital platforms can be used through targeted social media micro-campaigns (platform-specific content calendars), trusted local influencers, interactive Q&A sessions with clinicians/veterinarians, and short reminders timed to high-risk seasons. Simple mobile tools (or web forms) can facilitate reporting of animal abortions and high-risk exposures, provide step-by-step biosafety guidance, and route users to nearby services for testing and counseling.

Supplementary Table S1: Illustrative priority actions, implementation partners, and candidate monitoring indicators informed by observed brucellosis-related KAP gaps in Najran.

Priority action	Primary gap addressed (from this survey)	Lead stakeholder	Key partners	Candidate monitoring indicators

Tailored risk communication for diverse urban and peri-urban communities.	Informal sources like family and friends are the primary information channels at 53.9%, while health professionals provide limited guidance at 7.9%, and internet/media sources account for 3.3–4.6%. Additionally, 56.6% of individuals consume unpasteurized milk or dairy products.	Public health authorities / health cluster	Primary care, community nursing, Najran University, media channels, and community leaders	Reach; message recall; changes in KAP scores (repeated or pre/post when possible); percentage citing health professionals as information sources; brucellosis-related consultations or inquiries.
Hands-on training for occupations with higher exposure risks, such as farmers, slaughterhouse workers, and dairy vendors.	PPE gaps were observed during the handling of abortive materials, with gloves at 50.7% and masks at 38.8%, alongside inconsistent safe handling practices.	Animal health authorities / veterinary services	Municipality, slaughterhouses, farmer organizations, and veterinary services	% trained; PPE availability and use (via audit or observation); adherence to safe-handling protocols (checklist); and referrals or reporting to veterinarians.
Improve abortion-event management: reporting, segregation & safe disposal	Reported risky disposal behaviors included feeding aborted fetuses to dogs (14.5%) and discarding aborted materials in streets (11.2%), with 41.4% contacting a veterinarian and 32.2% segregating aborted animals.	Animal health authorities / veterinary services	Municipality, smallholder farms, and veterinary clinics	Abortion reports; percentages of safe disposal methods (such as burial or incineration according to local policy); rates of segregation and disinfection measures; and vaccination coverage (if available).
Food-chain risk reduction for raw milk and dairy products	Unpasteurized milk/dairy consumption was common (56.6%)	Food safety and municipal authorities	Ministry of Health, dairy outlets/vendors, community leaders	Inspections; percentage of outlets complying with pasteurization/labeling; percentage of households boiling or pasteurizing; prevalence of unpasteurized consumption in repeat surveys.
Standardize clinical counseling, testing pathways & case notification (where applicable)	Health professionals were rarely mentioned as an information source (7.9%), despite the presence of multiple self-reported brucellosis-related risk behaviors.	Public health authorities / health cluster	Hospitals, laboratories, IPC teams, and primary care	Clinician training coverage, percentage of febrile patients with documented exposure history, testing volume for suspected cases, turnaround times and completeness and timeliness of notifications.
One Health coordination & routine feedback (governance/learning loop)	Cross-sector coordination is needed to align information, food-chain and animal-abortion actions (not assessed in this survey)	Cross-sector coordination group (where established)	Public health authorities, animal health authorities, municipal authorities, and academic partners	Mechanism established (yes/no); joint review meetings per quarter; joint summary brief(s); action completion rate for agreed priorities
